# Characterization of Natural Antisense Transcript, Sclerotia Development and Secondary Metabolism by Strand-Specific RNA Sequencing of *Aspergillus flavus*

**DOI:** 10.1371/journal.pone.0097814

**Published:** 2014-05-21

**Authors:** Xinliang Wu, Bin Zhou, Chao Yin, Yong Guo, Ying Lin, Li Pan, Bin Wang

**Affiliations:** School of Bioscience and Bioengineering, South China University of Technology, Guangzhou, Guangdong, China; University of Cambridge, United Kingdom

## Abstract

*Aspergillus flavus* has received much attention owing to its severe impact on agriculture and fermented products induced by aflatoxin. Sclerotia morphogenesis is an important process related to *A. flavus* reproduction and aflatoxin biosynthesis. In order to obtain an extensive transcriptome profile of *A. flavus* and provide a comprehensive understanding of these physiological processes, the isolated mRNA of *A. flavus* CA43 cultures was subjected to high-throughput strand-specific RNA sequencing (ssRNA-seq). Our ssRNA-seq data profiled widespread transcription across the *A. flavus* genome, quantified vast transcripts (73% of total genes) and annotated precise transcript structures, including untranslated regions, upstream open reading frames (ORFs), alternative splicing variants and novel transcripts. We propose natural antisense transcripts in *A. flavus* might regulate gene expression mainly on the post-transcriptional level. This regulation might be relevant to tune biological processes such as aflatoxin biosynthesis and sclerotia development. Gene Ontology annotation of differentially expressed genes between the mycelia and sclerotia cultures indicated sclerotia development was related closely to *A. flavus* reproduction. Additionally, we have established the transcriptional profile of aflatoxin biosynthesis and its regulation model. We identified potential genes linking sclerotia development and aflatoxin biosynthesis. These genes could be used as targets for controlled regulation of aflatoxigenic strains of *A. flavus*.

## Introduction

*Aspergillus flavus* is a ubiquitous pathogenic fungus that infects plants and animals. Recently, studies of *A. flavus* gained tremendous attention owing to its health impact on agricultural commodities and related fermented products induced by aflatoxin contamination. Initially, studies using *A. flavus* as a model organism have been focused mainly on the aflatoxin biosynthesis pathway and on mechanisms of regulation of aflatoxin formation [1]–[5]. The highly negative impact of infection of agriculturally relevant plants by *A. flavus* caused gradual expansion of studies into the related fundamental areas of biology of this fungus, including the *A. flavus* secondary metabolism, sclerotia morphogenesis and propagation [2], [4], [6]–[9]. The recently revealed genomic sequence of *A. flavus* NRRL3357 provided a powerful tool for detailed analysis of biology of this fungus [10].

Sclerotia morphogenesis is an physiological process important for *A. flavus* propagation and involves various secondary metabolism pathways, including aflatoxin biosynthesis. Sclerotia are pigmented, specialized structures composed of compact *A. flavus* mycelia, which makes *A. flavus* resistant to harsh environmental conditions. Sclerotia likely derive from cleistothecia and might represent a vestige of sexual ascospore production [6]. According to the size of sclerotia, *A. flavus* could be divided into two groups: the L strain has sclerotia that are >400 µm in diameter and the S strain has sclerotia that are <400 µm in diameter [11], [12]. The S strains produce greater amounts of sclerotia and aflatoxin compared to the L strains under the same conditions of medium and cultivation [13], suggesting sclerotia morphogenesis and aflatoxin biosynthesis are closely related. To interpret this correlation, Chang and colleagues proposed the “substrate (acetate) competition” hypothesis. In their proposal, the increased production of aflatoxin results in a progressive decrease in sclerotial size, alterations in sclerotial shape and causes weakening of the sclerotial structure [6]. Recent efforts provided insights into the regulation of sclerotia morphogenesis. Ammonium, light, oxidative stress, temperature, organic acids and endogenous levels of cAMP might influence sclerotia formation and maintenance [14]–[19]. It was reported that *A. flavus laeA* and *veA* mutants are not able to form sclerotia [20], [21]. Cary and colleagues used DNA array to analyze sclerotia-related genes by comparing the *A. flavus* wild type strain and a *veA* mutant strain [22]; however, a detailed comprehensive description of sclerotia morphogenesis is lacking.

Natural antisense transcripts (NATs), a subset of non-coding RNAs (ncRNAs), are endogenous RNA molecules transcribed from the opposite DNA strand and can be complementary to the sense RNA through base pairing [23]–[25]. Expressed sequence tag (EST) sequencing, tiling microarrays, SAGE libraries, asymmetric strand-specific analysis of gene expression and global run-on sequencing (GRO-seq) could be used to identify NATs [26]. NATs are involved in transcriptional and post-transcriptional gene regulation by RNA interference (RNAi) [27], [28], chromatin-level gene silencing [23], [26], [29]–[31], chromatin remodeling [28], [32] and local chromatin modifications [33]–[35]. Global analysis of NATs has been done for mammals [23], insects [36], worms [37] and plants [38], [39]. It was reported that a large portion of the mammalian genome could produce transcripts from both strands [23], [40], [41]. For instance, >70% of mouse transcripts contain NATs and, owing to their prevalence, NATs are treated as pervasive features of mammalian genomes [26]. *Aspergillus* spp. have beenused as model organisms to investigate many molecular processes that govern the life of eukaryotic cells. Moreover, *Aspergillus* spp. have been used extensively to study fungal-specific pathways. NATs have been found in a divergent group of fungi, including the ascomycetes *Saccharomyces cerevisiae*, *Candida albicans*, *A. flavus*, *Magnaporthe oryzae, Tuber melanosporum* and *Schizosaccharomyces pombe,* and the basidiomycetes *Cryptococcus neoformans*, *Ustilago maydis* and *Schizophyllum commune* [28]. There are two distinct drawbacks in the study of *A. flavus* NATs, however; first, there were only 352 NATs (2.8% of the total open reading frames (ORFs)) found in *A. flavus* by analyzing ∼23,000 *A. flavus* cDNAs from cells grown under different nutritional conditions [25], while this number soars to 16.7–85.2% in comprehensively analyzed transcriptomes such as *S. cerevisiae* (16.7%), *C. albicans* (40.0%) and *S. pombe* (85.2%) [28]. Second, the biological function of NATs in *A. flavus* remained elusive due to the limited amount of NATs detected [42].

Owing to the importance of transcriptional regulation in the development of fungi, transcriptome profiling could be a valuable tool for establishing an in-depth understanding of *A. flavus* biology. The transcriptome of *A. flavus* has been studied by several groups, but these studies were not focused on sclerotia development or secondary metabolism. ESTs and microarrays have been used to identify genes involved in aflatoxin production using 7218 unique ESTs and microarrays containing more than 5000 unique *A. flavus* gene amplicons [1]–[3]. However, only 263 genes were expressed differentially and only 20 of the 29 aflatoxin pathway genes were identified owing to the limitation of microarrays in detecting genes with low levels of expression. Even an important transcriptional factor in the aflatoxin biosynthesis pathway, *aflR*, has not been detected [1]. Chang *et al* identified 22 features common to the aflatoxigenic/non-aflatoxigenic pairs by cross comparison. Possible roles of these identified genes have been discussed in relation to the regulation of aflatoxin biosynthesis [3]. Yu *et al* profiled the transcriptome of *A. flavus* under various temperature conditions in order to understand the effect of temperature on mycotoxin biosynthesis using RNA-seq technology. Only 23–29% of the total reads were mapped to genes, however, and >50% of the reads were mapped to rRNA genes and mitochondria and, therefore, were regarded as useless data [4].

We used strand-specific RNA sequencing (ssRNA-seq) to obtain an extensive transcriptome profile of *A. flavus* that might facilitate comprehensive understanding of the physiological processes of *A. flavus*. ssRNA-seq technology has all of the advantages of conventional RNA-seq technology, including low detection background, high dynamic detection range, high reproducibility and precise definition of transcript structure [43]. Furthermore, in contrast to conventional RNA-seq technology, ssRNA-seq data contain RNA polarity information and could decode a complex eukaryote transcriptome, including genome annotation, *de novo* transcriptome assembly and accurate digital gene expression analysis [44], [45]. ssRNA-seq technology has been used to parse the transcriptome of many organisms, including *S. cerevisiae* [46], *Mycoplasma pneumonia* [47], *Mus musculus* [47] and *Oryza sativa* [48]. By using ssRNA-seq data of the *A. flavus* mycelia and sclerotia cultures, we profiled genome-scale transcription, annotated precise transcript structure and provided in-depth depiction of *A. flavus* NATs. Analysis of differentially expressed genes might contribute to explicating sclerotia development and aflatoxin biosynthesis. In summary, our ssRNA-seq-based annotation provided a more extensive depiction of the *A. flavus* transcriptome and might contribute to the reduction of detrimental effects of *A. flavus* infection on agriculture.

## Materials and Methods

### Strain and Culture Conditions

*A. flavus* CA43 was kindly provided by Professor Perng-Kuang Chang (Southern Regional Research Center, Agricultural Research Service, U.S. Department of Agriculture, Washington, DC, USA). *A. flavus* CA43 belongs to the S strain isolates, which can produce numerous sclerotia and high amounts of aflatoxin [13].

Potato dextrose agar (PDA) medium (20 g of dextrose, 15 g of agar and the infusion from 200 g of potatoes per 1 L of medium, pH = 6.0) was used for *A. flavus* cultivation. For harvesting the *A. flavus* culture, a layer of cellophane was placed over the PDA medium plate (PDA-cellophane plate). *A. flavus* sclerotia (1.2×10^6^) were inoculated onto each PDA-cellophane plate and cultivated at 30°C in darkness. For RNA sequencing, the *A. flavus* mycelia were harvested after 48 h of cultivation (Aflavus_CA43_M) and sclerotia were collected after 7 days of cultivation (Aflavus_CA43_S).

### Strand-specific RNA-seq Library and Sequencing

The total RNA of each sample was extracted using RNAiso™ Plus (TaKaRa, Japan), treated with RNase-free DNase I (TaKaRa, Japan) and purified with a NucleoSpin® RNA Clean-up Kit (Macherey-Nagel, Germany). RNA integrity was analyzed by an Agilent Technologies 2100 Bioanalyzer. All samples had an RNA integrity number (RIN) >7.

The strand-specific RNA-seq library was prepared essentially as described but with some modifications [49]. Sera-mag magnetic oligo(dT) beads were used to isolate poly(A) mRNA from 20 µg of purified total RNA. mRNA was eluted by 10 mmol/L Tris buffer and fragmented into small pieces in the range of 100–500 bp by treatment with divalent cations. Taking these short fragments as templates, random hexamer primers were used to synthesize the first-strand cDNA using SuperScript II, RNaseH and DNA polymerase I. The first-strand cDNA was purified using a QIAquick PCR Purification Kit and used as the template for synthesizing dUTP-containing second-strand cDNA by adding buffer, dNTPs with dTTP replaced by dUTP, RNAseH and DNA polymerase I. Double-stranded cDNA fragments were purified with a QIAquick PCR Purification Kit and eluted with elution buffer for end-repair, phosphorylation and 3′-adenylation. After that, cDNA fragments were purified with a Qiagen MinElute PCR Purification Kit. Illumina sequencing adapters were ligated to the 3′-adenylated cDNA fragments followed by purification using a MinElute PCR Purification Kit. Fragments of 100–500 bp were purified using a QIAquick Gel Extraction Kit from the modified double-stranded cDNA fragments described above separated by TAE-agarose gel electrophoresis (2% (w/v) agarose; Certified Low-Range Ultra Agarose, Biorad). Uracil-DNA glycosylase (UNG) was added to digest dUTP-containing second-strand cDNA in an alkalescent medium at high temperature. The remaining first-strand cDNA was purified using a MinElute PCR Purification Kit and enriched by 15 rounds of PCR amplification using primers P7 and P5, which were homologous to the Illumina sequencing adapters. After purification using a QIAquick Gel Extraction Kit, fragments of 100–500 bp (average 200 bp) were selected as a strand-specific RNA-seq cDNA library. Finally, after quantification on an Agilent Technologies 2100 Bioanalyzer, the cDNA library was sequenced on the Illumina HiSeq2000 platform by a 90 bp paired-end sequencing strategy (using primers P7 and P5). The raw Illumina sequencing data for the *A. flavus* CA43 transcriptome was deposited in the SRA (http://www.ncbi.nlm.nih.gov/sra/) with ID no.SRP018670.

### Primary Splitting of Raw Strand-specific Sequencing Data

In the course of strand-specific paired-end (PE) sequencing, reads consistent with the reverse strand of a transcript were first obtained by primer P5-triggered sequencing (read1), and reads consistent with the forward strand of the same transcript were then obtained by primer P7-triggered sequencing (read2). Read1 and read2 are PE reads.

According to strand-specific sequencing features, read1 mapped to the forward strand of a transcript and read2 reversely mapped to the forward strand of the transcript could not reflect the real transcription and was discarded. The remaining mapping reads were used to analyze gene expression and genes expressed differentially.

As the forward strand of a gene might be located in either the forward or the reverse strand of the genome, we classified read1 reversely mapped to the forward strand of the genome and read2 mapped to the forward strand of the genome into one group representing transcription from the forward strand of the genome. We classified read1 mapped to the forward strand of the genome and read2 reversely mapped to the forward strand of the genome into a group representing transcription from the reverse strand of the genome. The mapping result with genome sequence was used for the following analysis, including novel transcript predication, untranslated region (UTR) analysis and alternative splicing (AS) events. This splitting strategy was designed according to the BGI analysis pipeline (http://www.genomics.cn/index).

### Read Mapping and Normalization of Gene Expression

The reference genome (*A. flavus* NRRL3357) was downloaded from GenBank (accession no. AAIH00000000.2) and the gene annotation data were downloaded from the AspGD website (http://www.aspergillusgenome.org/download/sequence/A_flavus_NRRL_3357/current/). After removing the reads containing sequencing adapters and low-quality reads (reads containing Ns >9), the remaining 90 bp clean reads were aligned to the *A. flavus* reference genome and annotated genes using SOAP2 software [50], allowing up to five base mismatches for genome alignment and three base mismatches for gene alignment. Reads that failed to be mapped were first trimmed off 1 base from the 5′-end. If there was still no match, these reads were trimmed off progressively two bases each time from the 3′-end and mapped to the genome until a match was found (unless the read was trimmed <48 bases for genome alignment or <28 bases for gene alignment). The insert between PE reads was set to 1 bp-10 kb, allowing them to span introns or the intergenic regions of various size in the genome. When PE reads were mapped to non-redundant genes, the insert was set to 1 bp-1 kb. The coverage per base in the genome and the reads count covering each transcript could be calculated by reads mapping.

We used the popular RPKM method to normalize the transcript level, which was expressed as the number of reads per kilobase of exon region per million mapped reads (RPKM) [51], [52]. The cutoff value for gene transcriptional activity was determined on the basis of the 95% confidence interval (CI) for all RPKM values. GO annotation of the *A. flavus* genes was done by Blast2GO (version 2.6.3) software [53] and visualized by WEGO software [54].

### Non-redundant EST Sequence

Non-redundant ESTs were obtained by mapping all *A. flavus* EST sequences (http://compbio.dfci.harvard.edu/cgi-bin/tgi/est_ann.pl?gudb=a_flavus) to *A. flavus* annotated genes using BLAT software [55], [56]. ESTs that could not be mapped to annotated genes (http://www.aspergillusgenome.org/download/sequence/A_flavus_NRRL_3357/current/) were selected as non-redundant ESTs.

### UTR and Upstream Open Reading Frame Analysis

UTRs were defined as regions flanking a gene coding sequence, with contiguous expression of each base supported by at least two uniquely mapped reads. Transcripts whose ends overlapped with each other were discarded. The length of 5′- or 3′-UTRs was limited to a maximum length of 1000 bp. The upstream ORFs (uORFs) were searched for in the 5′-UTRs of genes. The length of uORFs was set to 9–150 bp, and the distance between a uORF and a gene start codon should be <500 bp.

### Detection of Novel Transcripts

Novel transcriptionally active regions (TARs) were determined in the intergenic regions by contiguous expression of each base supported by at least two uniquely mapped reads and length >35 bp [43]. A novel TAR should be separated by at least 200 bp from the upstream or downstream region of the transcript. A novel transcript is composed of novel TARs connected by at least one paired read. Novel transcripts of length >150 bp were selected for further analysis.

### Alternative Splicing Events in *A. flavus*

Potential junction sites were detected using the TopHat program [57]. Reads used to identify junction sites could not map to the genome but were able to map to the genome after several terminal bases were trimmed off. An acknowledged junction site should be supported by at least two mapped reads with different mapping positions within the junction site region and with a minimum of 5 bp mapping on each side of the junction site and a tolerance of 2 bp mismatch [43]. A novel junction site should be supported by at least four such reads. Using the method described by Wang *et al* [58], seven types of AS events were analyzed in *A. flavus* CA43, including skipped exons (SE), retained introns (RI), alternative 5′-splice sites (A5SS), alternative 3′-splice sites (A3SS), mutually exclusive exons (MXE), alternative first exons (AFE) and alternative last exons (ALE).

### Natural Antisense Transcript

Putative antisense transcripts were detected for *A. flavus* annotated genes with RPKM >33. For a certain annotated transcript, the corresponding antisense transcript was denoted as a complementary overlapping region on the opposite strand with contiguous expression of each base supported by at least one uniquely mapped read and length >200 bp. In order to obtain reliable results, the detected antisense transcripts should have an average coverage depth >2. To eliminate the influence of an intronic region, antisense transcripts were analyzed in the transcriptional exons and UTRs.

### Analysis of Differentially Expressed Genes between *A. flavus* Mycelia and Sclerotia States

The DEGseq R package was used to analyze differentially expressed genes (DEGs) by reads count covering each gene on the basis of the Random Sampling model [59]. Functional enrichment analysis of certain DEGs was performed with Blast2GO software using Fisher’s exact test with robust false discovery rate (FDR) correction of 0.05.

## Results and Discussion

### Sequencing Summary

**Figure 1:**
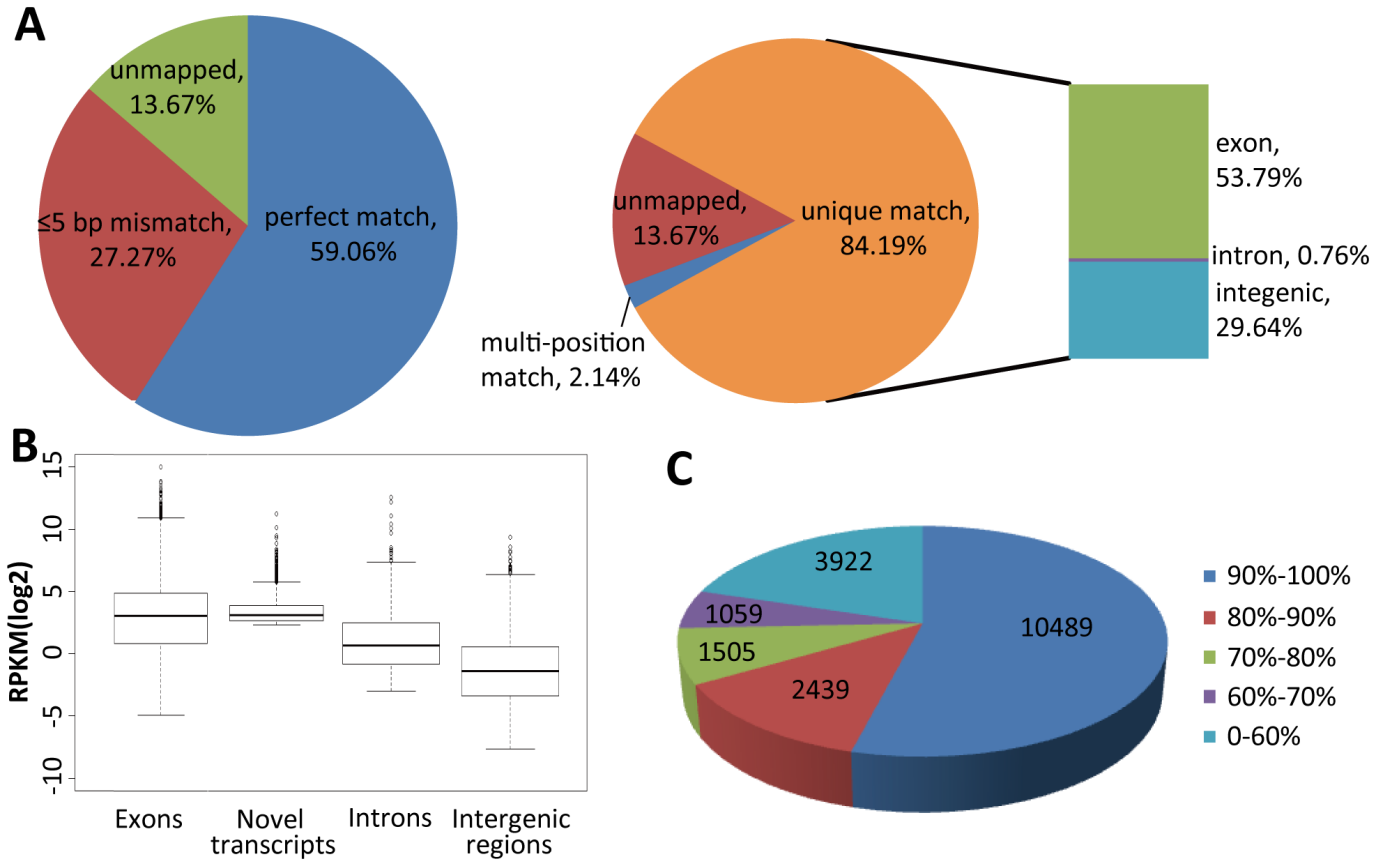
Summary of RNA sequencing of *A. flavus* CA43. (A) Matching summary of ssRNA-seq reads to the *A. flavus* genome. (B) Box and whisker plots of log_2_-transformed RPKM for exons, novel transcripts, introns and intergenic regions. Horizontal lines in boxes, the first, median and third quartile. Other horizontal lines, the inner boundaries. Diamonds, data outside the inner boundaries. (C) Mapping coverage of the transcribed *A. flavus* genes.

To obtain an elaborate transcriptome profile of *A. flavus*, the isolated mRNA of *A. flavus* CA43 mycelia (Aflavus_CA43_M) and sclerotia (Aflavus_CA43_S) were subjected to high-throughput sequencing by the strand-specific paired-end sequencing strategy. In total, 26.9 and 27.7 million of 90 bp Illumina reads were obtained for Aflavus_CA43_M and Aflavus_CA43_S, reaching an average genome coverage depth of 60-fold and 62-fold, respectively. About 85% of all reads were mapped uniquely to the *A. flavus* genome with a tolerance of 5 bp mismatch (84.48% for Aflavus_CA43_M and 83.91% for Aflavus_CA43_S; **Fig. 1A** **and Table S1**). Approximately 55% of all reads were mapped uniquely to annotated *A. flavus* genes (**Table S1**), which represents much greater accuracy compared to earlier *A. flavus* transcriptome data (23–29%) [4]. The 30% of reads mapped uniquely that could be mapped to the genome but could not be mapped to the annotated genes might represent novel transcripts hidden in the intergenic regions. Massive non-redundant ESTs in the *A. flavus* genome (**Table S2**) suggest the presence of a large number of novel transcripts. For all *A. flavus* transcripts, 38.34 million reads were mapped to their sense strand but only 0.65 million reads (1.7%) were mapped to their opposite strand, suggesting our RNA-seq library is strand specific. The reads mapped to the opposite strand might represent natural antisense transcripts that occur normally in eukaryotes. Moreover, the low ratio of reads mapped to introns suggests that our RNA-seq data could depict the transcription of *A. flavus* genomic loci precisely.

### Extensive Depiction of *A. flavus* Transcriptome by ssRNA-seq Data

The global transcriptional profile of the *A. flavus* mycelia culture (Aflavus_CA43_M) is shown in **Fig. S1**. About 67.55% of the *A. flavus* genome (39.91 Mb) was expressed as ssRNA-seq reads, quantifying the transcriptional abundance for 9871 *A. flavus* genes (73% of 13,487 genes) with a 95% CI (**Table S2**). However, the EST-based annotation assembled only 3749 tentative consensus sequences [2]. The genome coverage was comparable to that of *A. oryzae* RIB40 (76.66%) [43], and >50% of the expressed genes had a sequencing coverage of >90% (**Fig. 1C**). GO annotation showed that 7652 of the 9871 transcribed genes were assigned to GO categories (**Table S2**), providing rich information for the investigation of gene function. The expression levels of exons were much higher compared to introns and intergenic regions (**Fig. 1B**), and only very few introns (1621 of total 27,783 introns) were mapped by ssRNA-seq reads. Therefore, ssRNA-seq based annotation provided a more extensive depiction of the *A. flavus* transcriptome.

**Figure 2:**
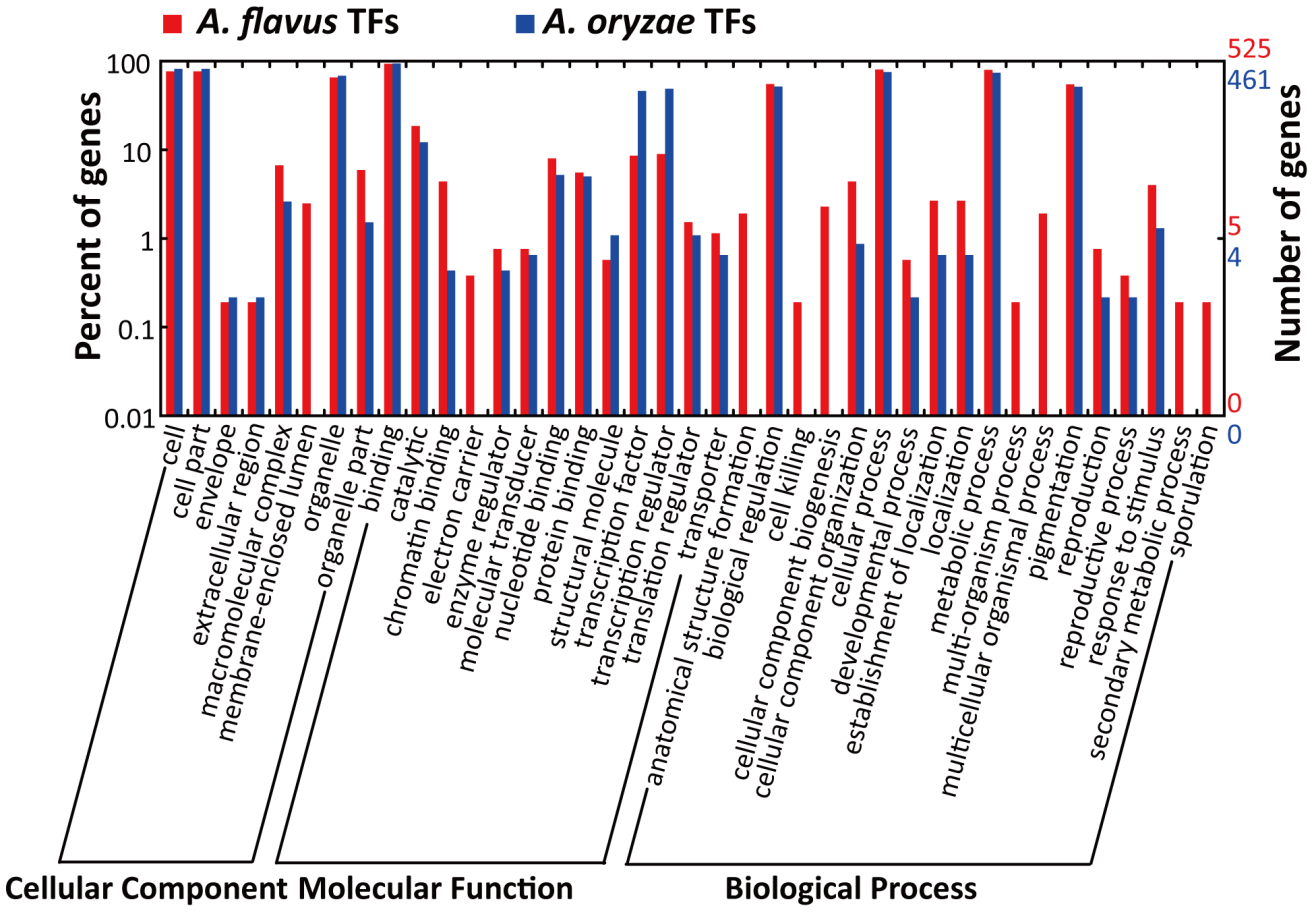
WEGO comparison of *A. flavus* and *A. oryzae* transcribed transcriptional factors. In total, 525 of the 617 *A. flavus* transcribed TFs and 461 of the 571 *A. oryzae* transcribed TFs were assigned to GO categories.

We analyzed the transcriptional activity of *A. flavus* transcriptional factors (TFs) to address extensive transcription of the *A. flavus* CA43 genome. Fungal TF information was downloaded from the web (http://ftfd.snu.ac.kr/tf.php?a=summary0&o=o). The *A. flavus* genome contains 667 TFs and 617 of them were expressed as RNA-seq reads, while *A. oryzae* contains 603 TFs and 571 of them were transcribed (**Table S3**; the data for *A. oryzae* TFs were obtained from our earlier work [43]). GO analysis showed *A. flavus* CA43 TFs were enriched mainly in the GO categories of binding, catalytic, biological regulation, nucleotide binding, cellular process and metabolic process (**Fig. 2**). Particularly, *A. flavus* CA43 contains transcribed TFs involved in secondary metabolic process and sporulation.

The non-homologous end joining (NHEJ) pathway is important for DNA repair and gene recombination. NHEJ-deficient strains could be used efficiently for gene targeting and homologous gene recombination [60]. *ku70*, *ku80* and *LigD* are the essential genes in the NHEJ pathway of *Aspergillus* spp. [61]. For *A. oryzae* RIB40, the transcriptional level of *ku80* (AO090026000842) reached RPKM 82.82 in the solid-state culture, and *ku70* (AO090701000906) and *LigD* (AO090113000208) were not expressed [43]. For *A. flavus* CA43, *ku70* (AFL2G_06276), *ku80* (AFL2G_06694) and *LigD* (AFL2G_08329) were all transcribed under both mycelia and sclerotia cultures (RPKM 23.57, 18.84 and 14.28 for mycelia culture, and 150.82, 31.34 and 231.45 for sclerotia culture) (**Table S2**), suggesting *A. flavus* CA43 possessed highly efficient non-homologous gene targeting.

### Improved Annotation of *A. flavus* Gene Models

**Figure 3:**
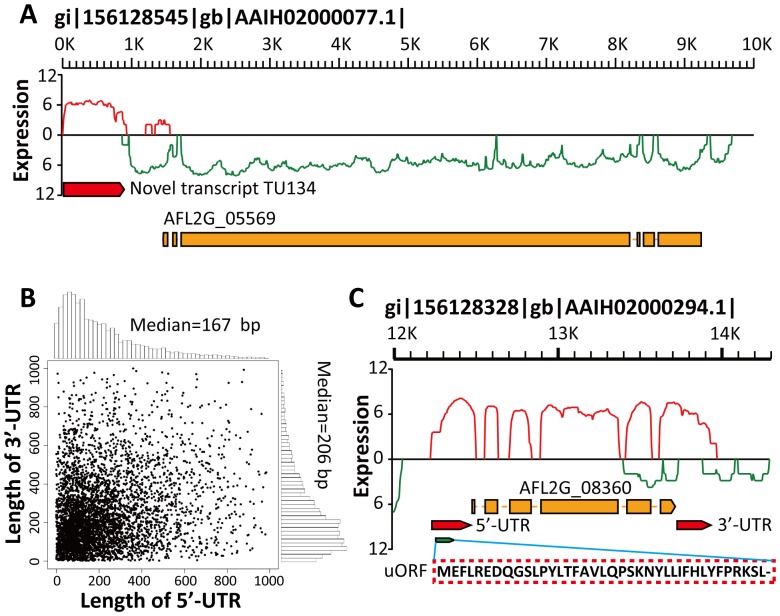
Detailed annotation of the *A. flavus* gene model. (A) A novel transcript (TU134) identified in contig gi|156128545|gb|AAIH02000077.1| in the forward orientation. Red bar, the novel transcript TU134 identified in the mycelia sample. Red curve, expression level (log_2_-transformed reads count) of the transcripts located in the forward strand. Green curve, the expression level of the transcripts located in the reverse strand. (B) Scatterplot and histograms showing the length distribution of the 5′- and 3′-UTRs of *A. flavus* CA43 genes. (C) UTR and uORF illustration for an annotated *A. flavus* gene (AFLG2_08360).

Besides the reads mapped to the 13,487 annotated *A. flavus* genes, 30% of reads mapped uniquely were located in the intergenic regions. Extensive mapping and clustering of these intergenic reads revealed 939 and 1196 previously unrecognized novel transcripts for mycelia and sclerotia samples, respectively (**Table S4**). The levels of expression of these novel transcripts were almost as high compared to exons (**Fig. 1B**) and 62.62% of the identified novel transcripts were longer than 500 bp (**Table S4**). This ratio is much higher compared to *A. oryzae* RIB40 [43]. Most of the identified novel transcripts were non-coding RNAs (ncRNAs) and only 25 (1.17%) novel transcripts were predicted to be potential protein-coding genes with an ORF length of ≥150 bp. The vast majority of transcribed ncRNAs might act as NATs to regulate gene expression. One of the identified novel transcripts (TU134) in mycelia culture is illustrated by **Fig. 3A**, with a length of 848 bp and an average sequencing depth of 11.61.

The transcribed genome sequence assembled by our ssRNA-seq reads could be used to define or extend untranslated regions (UTRs) of the gene, which have important roles in post-transcriptional regulation [62]. The 5′-UTRs for 5994 transcripts and the 3′-UTRs for 6407 transcripts were determined in this study (**Table S5**). Most (88.87%) of these identified UTRs were <500 bp with a median length of 167 bp for 5′-UTRs and 206 bp for 3′-UTRs (**Fig. 3B**). The median length of UTRs in *A. flavus* CA43 was longer compared to *A. oryzae* RIB40 (107 bp for 5′-UTRs and 156 bp for 3′-UTRs) [43] and *Schizosaccharomyces pombe* (152 bp for 5′-UTRs and 169 bp for 3′-UTRs) [63]. According to the study reported by Lackner *et al* suggesting the most stable transcripts have short 5′-UTRs and the least stable transcripts have long 5′-UTRs [64], *A. flavus* CA43 has less stable transcripts and a much higher RNA turnover rate compared to *A. oryzae* and *S. pombe*. It was reported that ORFs in 5′-UTRs upstream of annotated start codons (uORFs) might constitute important regulatory factors for gene expression [65], [66]. We have predicted 2600 uORFs for *A. flavus* annotated genes (19.28%; **Table S5**), which is much higher compared to *A. oryzae* RIB40 (11.14%) [43] and *S. cerevisiae* (6%) [67]. An example of a uORF and the corresponding 5′-UTR is shown in **Fig. 3C**. Genes containing uORFs are enriched specifically for GO terms of cellular protein modification process, protein serine/threonine kinase activity, Ras GTPase activator activity, positive regulation of Ras GTPase activity, phosphorylation, autophagy, phospholipid binding and protein transport (FDR-adjusted *p*<0.05; **Fig. S2 and Table S5**).

**Figure 4:**
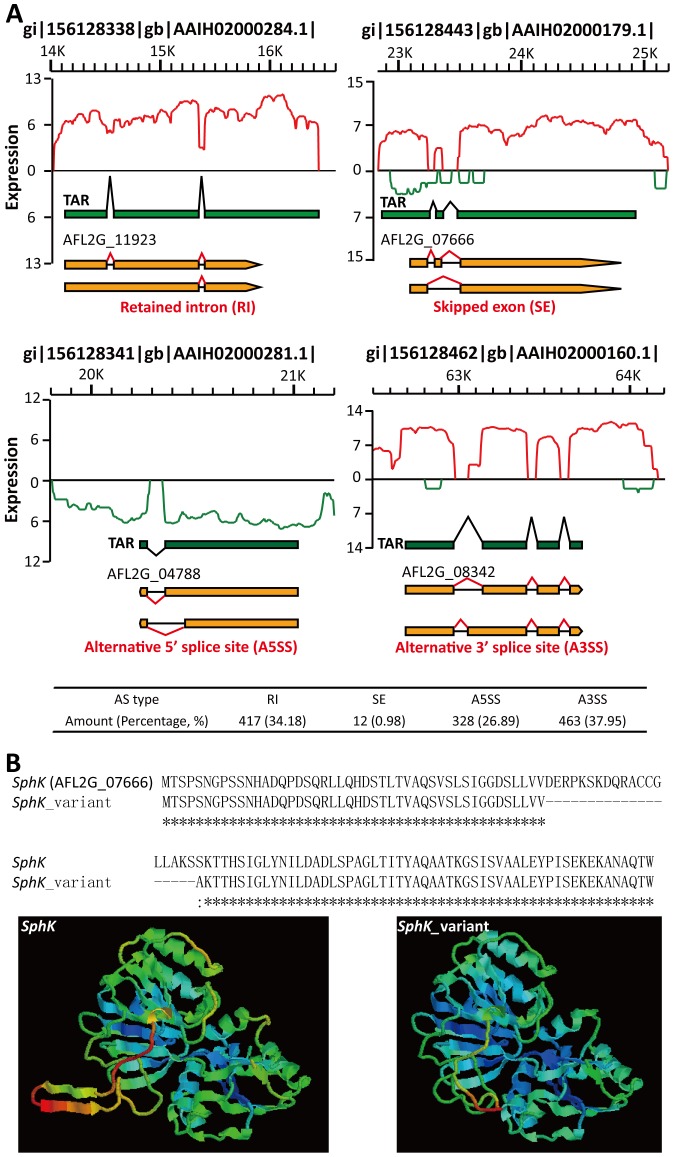
Illustration of AS events (RI, SE, A5SS and A3SS) identified in the *A. flavus* transcriptome. (A) Green bar, the transcriptional active region (TAR). Orange bar, *A. flavus* annotated genes. Knuckle lines (black or red), the relation with TARs or exons. The types and amounts of AS events are shown. (B) Amino acid alignment and homologous 3D modeling of AFL2G_07666 (*SphK*) and its AS variant (*SphK* _variant). Broken line, the skipped exon of *SphK*.

AS events contribute to producing multiple proteins from genes with two or more exons in fungi. This could enrich the proteomic diversity of fungi and provide the ability to survive in a hazardous environment. AS events were analyzed in 9545 *A. flavus* multi-exon genes (70.77% of all *A. flavus* genes) using the method described by Wang *et al* [58]. A total of 1220 AS events took place in 941 *A. flavus* genes (**Table S6 and** **Fig. 4A**), including retained introns (RIs), skipped exons (SEs), alternative 5′-splice sites (A5SSs) and alternative 3′-splice sites (A3SSs). About 12.78% of the *A. flavus* multi-exon genes produced AS isoforms, similar to *A. oryzae* RIB40 (11.10%) [43] and many more than *Pichia pastoris* (4.78%) [68]. This is in agreement with the conclusion that the frequency of AS events is proportional to the ratio of multi-exon genes in a genome (76.98% for *A. oryzae* and 11.91% for *P. pastoris*) [69]. AS events might alter the amino acid composition and the structure of the target protein. For example, AFL2G_07666 (**Fig. 4A**), encodes sphingosine kinase (*SphK*), which participates in the sphingosine 1-phosphate (S1P) metabolism. Together with S1P phosphatase (*S1PP*) and S1P lyase (*SPL*), AFL2G_07666 controls the intracellular S1P level and has important roles in the regulation of cell migration, survival, differentiation, angiogenesis and development through an extracellular signaling pathway mediated by a family of specific G protein-coupled receptors. We performed homologous modeling using SWISS-MODEL Workspace [70], [71] and constructed a 3D model of the AFL2G_07666 transcript and its AS variant (**Fig. 4B**). The skipped exon alters the 3D structure of AFL2G_07666 and might influence its biological function. To investigate the mechanism of AS events, we calculated the ratio of the amount of RIs to cassette exons (CEs, including SE, AFE, ALE and MXE). The high RI/CE ratio (34.75) indicates *A. flavus* might recognize splicing sites and produce AS events mainly by the intron definition (ID) mechanism, according to the study reported by McGuire *et al* [69].

### Natural Antisense Transcript

The “G-value paradox”, that the amount of protein-coding genes does not correlate with the complexity of an organism, suggests that so-called junk DNA in a genome contains rich regulatory information exerting via transcribed natural antisense transcripts (NATs) [23], [28]. So far, little is known about the role of NATs in *Aspergillus* spp. It was suggested that NATs might have an important role in differential expression of genes involved in secondary metabolism in *A. oryzae* [42]. According to an earlier study, the most prominent NAT type is the non-protein-coding antisense RNA partner of a protein-coding gene [26]. We searched for NATs in our data for all *A. flavus* protein-coding genes with the RPKM>33 using stringent criteria. In all, 1124 and 839 NATs were identified in the AflavusCA43_M and AflavusCA43_S samples, respectively (**Table S7**). This is many more compared to *A. flavus* NATs on the basis of EST data (352, 2.8%) and *Cryptococcus neoformans* NATs (53, 0.8%) [25]. The number of RNA-seq-based *A. flavus* NATs is in the same range as *S. cerevisiae* (1103, 16.7%) [46]. However, this frequency is much lower compared to *Candida albicans* (2458, 40%) and *M. oryzae* (4215, 32.8%) [28].

**Figure 5:**
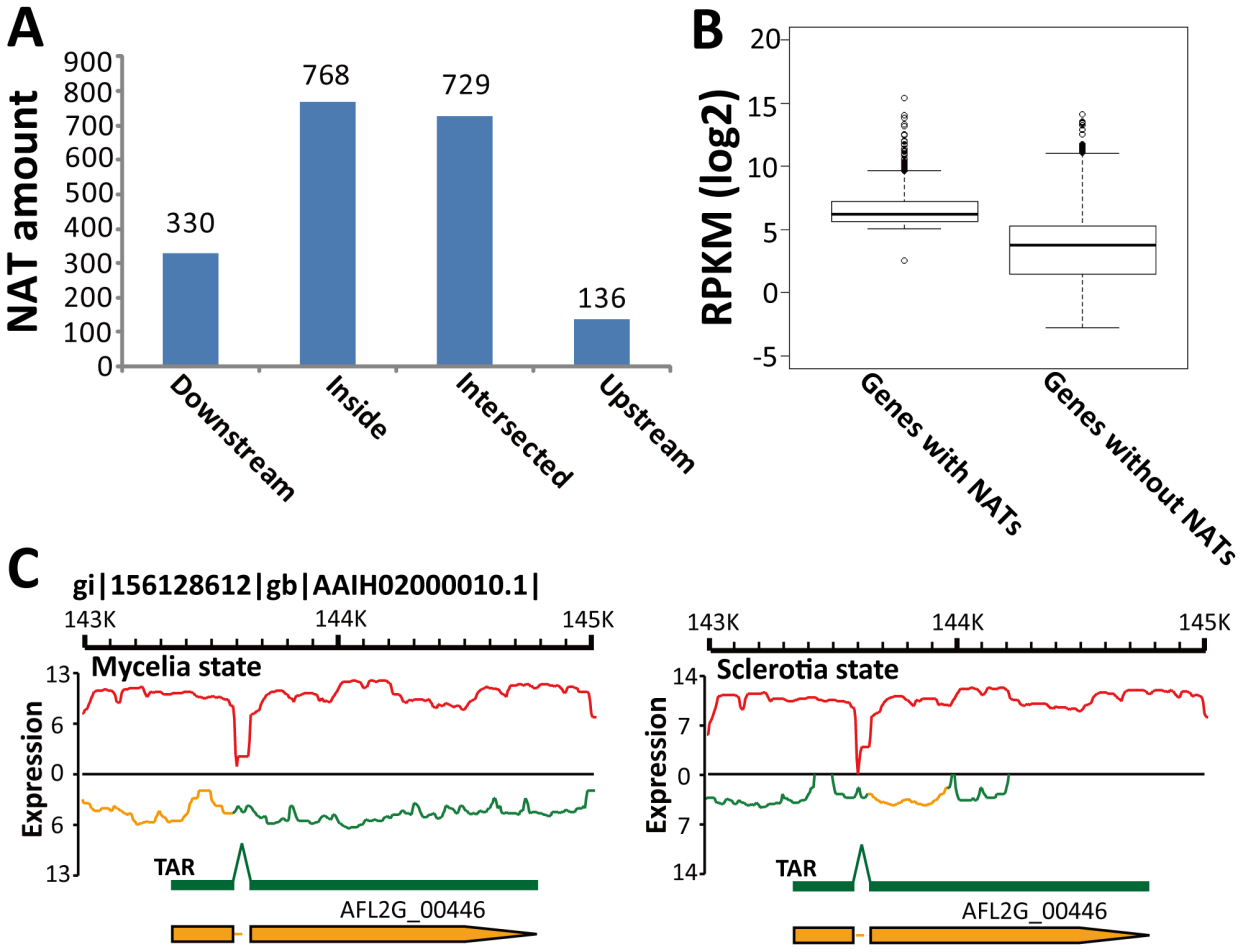
NAT analysis in *A. flavus* CA43. (A) The relationship between the location of NAT and its corresponding genes. Downstream, Upstream and Inside, NATs located in the downstream, upstream or inside of their corresponding genes, respectively. Intersected, NAT located across one gene and its 5′- or 3′- flanking region. (B) Box and whisker plots of log_2_-transformed RPKM for *A. flavus* annotated genes with or without NATs. (C) Occurrence of NAT in the *SdeA* gene (AFL2G_00446). Orange curves, the NAT in the *SdeA* gene.

There are more NATs located inside coding regions (768, 39.14%) compared to UTR regions (466, 23.76%, **Fig. 5A**). This means NATs in *A. flavus* might regulate gene expression mainly at the post-transcriptional level, which is consistent with the conclusion reported by Donaldson *et al* [28]. The inside-biased NAT distribution in *A. flavus*, however, was different from the 3′-biased antisense transcription in *A. nidulans* [72] and the mammalian NAT distribution, where NATs are usually enriched in the region of the 250 bp upstream sequence and the 1.5 kb downstream sequence [26]. The transcriptional level of *A. flavus* genes with NATs (average RPKM 253.59) was much higher compared to genes without NATs (average RPKM 54.50; **Fig. 5B**). This is consistent with the earlier study by Katayama *et al* [23], strongly denying the simple hypothesis that NATs are just negative regulatory elements and artifacts of leaky bidirectional transcription [26], [28], [73].

Among the 352 EST-based NATs in *A. flavus*, only 19 were verified by our RNA-seq data (**Table S7**). One of the EST-based NATs is that of the aflatoxin biosynthetic regulator gene (*aflR*, AFL2G_07224), which was identified in our study when the RPKM cutoff for NAT analysis was set at 5 (**Table S7**). The existence of a NAT in the transcriptional factor *aflR* suggests the interaction between a NAT and its sense transcript can occur in the nucleus, consistent with the conclusion from studies in mammals [23], [26]. Another example is *SdeA* (AFL2G_00446), which is involved in the regulation of morphology under temperature change and in the production of multicellular developmental structures (conidiophores, cleistothecia and sclerotia) [25]. Changes in the expression of *SdeA* and its NAT in the mycelia and sclerotia states suggests NAT participates in the regulation of *SdeA* and thus biological processes controlled by this gene (**Fig. 5C**).

GO enrichment analysis demonstrated genes with NATs were enriched specifically in GO terms of protein complex, protein binding, RNA binding, translation, ribosome, intracellular protein transport, cellular amino acid metabolic process, vesicle-mediated transport, hexose catabolic process and biological regulation (FDR-adjusted *p*<0.05; **Fig. S3 and Table S7**). Thus, these NAT-containing genes are closely related to protein expression, secretion and energy production in *A. flavus*. For example, the *A. flavus Set1* gene (AFL2G_02936) has a NAT located inside its coding sequence (**Table S7**). According to the report that the corresponding NAT could prevent *Set1*-mediated transcription initiation in *S. cerevisiae* [28], NAT might influence the *Set1*-mediated transcription initiation in *A. flavus*. The discussion about NAT function in fungi was focused mainly on the NAT-mediated alteration of physiological processes in response to environmental nutrient starvation [28] and nitrogen metabolism [72]. Our analysis is the first global investigation of NAT function in *A. flavus*.

### Sclerotia Development and Reproduction

Sclerotia are considered to derive from cleistothecia, which is the sexual reproductive organ in *Aspergillus* spp. [6]. Genes involved in sexual reproduction and the balance of sexuality and asexuality of *Aspergillus* spp. are given in **Table S8** [74]–[76]. To identify genes involved in *A. flavus* sclerotia development, differentially expressed genes (DEGs) between the *A. flavus* mycelia and sclerotia cultures were detected. For the 13,487 *A. flavus* genes, 9871 (73.19%) were transcribed under both conditions. A total of 661 genes were expressed specifically in the mycelia state and 343 genes were specific for the sclerotia state. These genes might represent factors critical for the physiological development of *A. flavus*. There were 7609 DEGs (56.42%) between the *A. flavus* mycelia and sclerotia states, with 1821 up-regulated genes and 5788 down-regulated genes in the sclerotia state (*p*<0.001; **Table S9**). DEGs between the *A. flavus* mycelia and sclerotia states are much more abundant compared to *A. flavus* cultivated at different temperatures (2709) [25], indicating different developmental stages are accompanied by a high level of diversity in gene expression.

**Figure 6:**
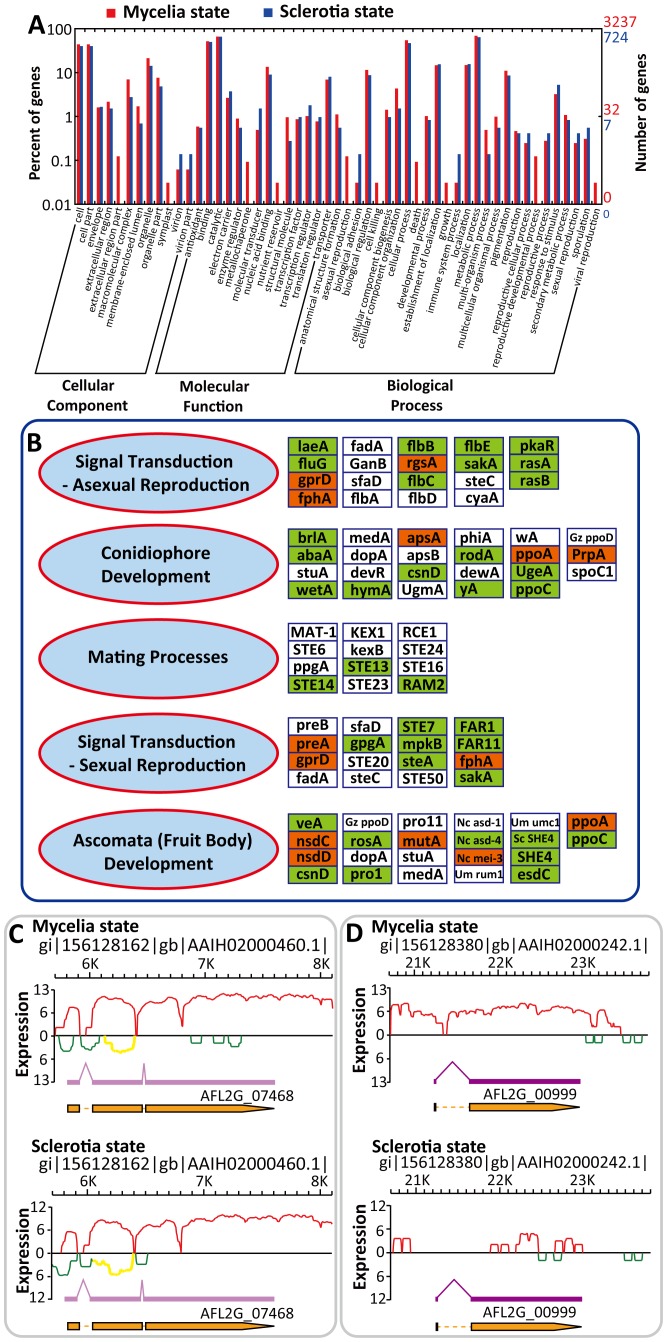
Genes expressed differentially between *A. flavus* mycelia and sclerotia states. (A) WEGO illustration of the up-regulated genes in the *A. flavus* mycelia and sclerotia states. In total, 3237 of the 4492 up-regulated genes in the mycelia state and 724 of 1149 up-regulated genes in the sclerotia state are assigned to GO categories. (B) The model of regulation of *A. flavus* reproduction-related genes in the mycelia and sclerotia states. Boxes, DEGs (*p*<0.001 and change >2-fold) between the mycelia and sclerotia states, denoted by the names of their homologs in *A. nidulans*, *S. cerevisiae* (Sc) and *Neurospora crassa* (Nc). Red and green boxes, the up-regulated and the down-regulated genes in the sclerotia state, respectively. White boxes, genes with no significant difference between the mycelia and the sclerotia states. (C) Occurrence of NAT in the *veA* gene (AFL2G_07468). Yellow curve, NAT in the *veA* gene. (D) Transcriptional status of the conidial transcriptional factor *brlA* (AFL2G_00999).

To identify genes closely related to the developmental stages, DEGs with change >2-fold were selected for further analysis: 1149 up-regulated genes and 4492 down-regulated genes were detected for the sclerotia state. WEGO illustration showed GO terms of the reproductive cellular process, the reproductive process, sexual reproduction and sporulation contained more up-regulated genes in the sclerotia state compared to the mycelia state (**Fig. 6A**), indicating sclerotia is closely related to *A. flavus* reproduction instead of being only a sexual vestige. Additionally, the abundance of residual mating process genes in *A. flavus* suggests it might be capable of sexual development.

DEGs related to reproduction are shown in **Fig. 6B**, with 14 up-regulated genes and 37 down-regulated genes in the sclerotia state. The zinc finger protein-encoding gene *brlA* (AFL2G_00999) is the primary activator of asexual conidiation reproduction [77], [78]. Down-regulated *brlA* in the sclerotia state (**Fig. 6D**), together with a series of conidiation-related genes (*abaA*, *wetA*, *flbB*, *flbC* and *flbE*; **Fig. 6B**), suggests conidial development is repressed during sclerotia development. These findings might contribute to understanding the sexual and asexual balance of *A. flavus*.

### Secondary Metabolism

The secondary metabolic (SM) pathways in fungi consist of many genes encoding polyketide synthetases (PKS), fatty acid synthases, dehydrogenases, reductases, oxidoreductases, epoxide hydrolases, cytochrome P450 monooxygenases and methyltransferases [1]. However, it is difficult to determine whether such genes are involved in SM pathways, because vast numbers of genes are categorized as belonging to the gene clusters mentioned above. On the basis of the web tool SMURF [9], 55 putative SM pathways were identified in *A. flavus*, including 22 PKS and 27 non-ribosomal peptide synthetase (NRPS) pathways. It is important to study secondary metabolism in *A. flavus* because it was reported that secondary metabolism was often related to sporulation and sclerotia development [2], [76]. In our RNA-seq data, backbones of 38 SM pathways were transcribed in both the mycelia and sclerotia cultures (**Table S10**), including the aflatoxin (AF) biosynthetic pathway (cluster #54).

**Figure 7:**
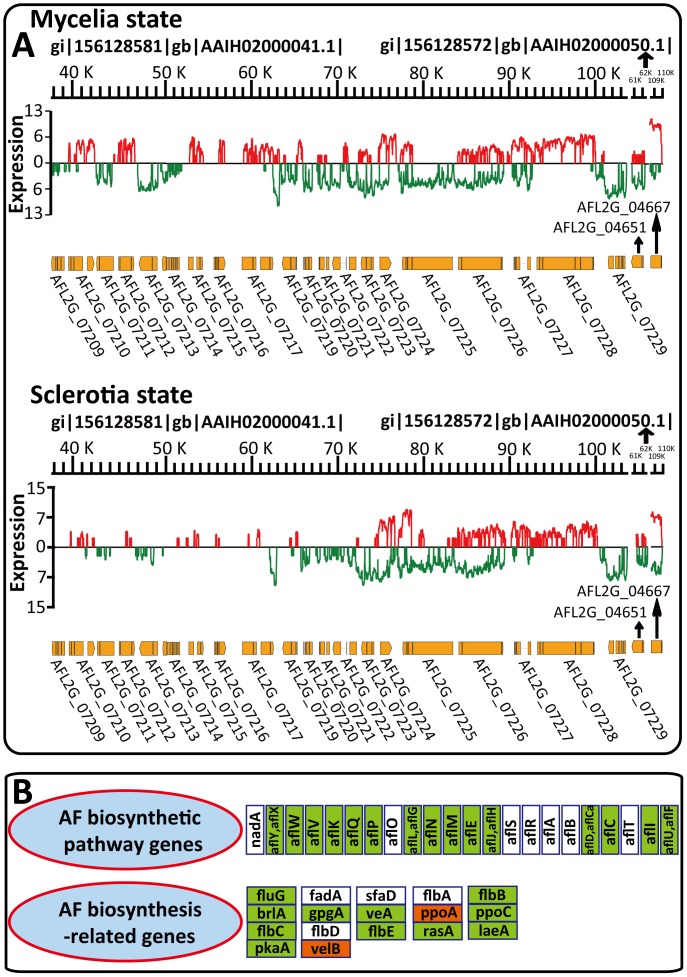
Differential transcription of *A. flavus* aflatoxin biosynthetic pathway. (A) The expression status of AF biosynthesis in the mycelia and sclerotia states. (B) The regulation model of AF biosynthesis-related genes in the mycelia and sclerotia states. The orthologs of the AF biosynthetic pathway genes are in the same order as in (A). Boxes, DEGs (*p*<0.001 and change >2-fold) between mycelia and sclerotia states, denoted by the names of their homologs in *A. nidulans*. Red and green boxes, up-regulated and down-regulated genes in the sclerotia state, respectively. White boxes, genes with no significant change between the mycelia and sclerotia states.

The AF biosynthesis pathway is the best studied SM pathway in *A. flavus*, containing at least 23 enzymatic reactions and 29 genes in a 75 kb cluster on chromosome III (**Table S10**) [79]. Only eight transcribed genes in the AF pathway were scored by microarray technology [1]. Our RNA-seq data provided precise information about AF biosynthesis. The transcriptional factor *aflR* (AFL2G_07224) was transcribed under both the mycelia and sclerotia conditions and the NAT of *aflR* was transcribed under the sclerotia condition, suggesting AF biosynthesis might be down-regulated by NAT-mediated RNAi during sclerotia development (**Table S10**). This is consistent with the fact that the transcripts of all AF pathway genes were detected in the mycelia state but three genes (*aflD*, *aflCa* and *aflP*) were not detected in the sclerotia state (**Table S10**). Additionally, changes in expression of the global SM transcriptional factor *veA* (AFL2G_07468) during sclerotia development suggest it has roles in linking sclerotia development with secondary metabolism (**Fig. 6C** **and Table S8**) [80]. These findings are in agreement with the transcriptional profile of AF biosynthesis (**Fig. 7A**) and the down-regulation of most AF biosynthetic structural genes in the sclerotia state (**Fig. 7B**).

Genes participating in the AF metabolic pathway contain biosynthesis genes, signal transduction genes, regulatory genes and genes involved in the stress response [2]. Although most of the AF structural genes are in a single cluster, this cluster might be regulated by genes spread throughout the *A. flavus* genome [81]. Among the AF-related genes given in **Table S10**, 11 were down-regulated in the sclerotia state and two were up-regulated (**Fig. 7B**). According to our analysis of sclerotia development (**Table S8 and** **Fig. 6B**), these AF genes were also related to sclerotia development, suggesting they link sclerotia development and aflatoxin biosynthesis. Therefore, high throughput RNA-seq data brought new insights into *A. flavus* mycotoxin production and other secondary metabolism pathways in this fungus. AF contamination of crops is a heavy economic burden on farmers and it is important, therefore, that the target genes identified by RNA-seq data can be used in the biocontrol of aflatoxigenic strains by genetic manipulation.

## Conclusions

The *A. flavus* transcriptome has been studied intensively by several research groups in recent years. Despite these efforts, however, the mechanism underlying the regulation of *A. flavus* physiology is unknown. Our data profiled the *A. flavus* transcriptome on the genomic scale and annotated transcript structures precisely. UTR annotation revealed *A. flavus* might have a much higher RNA turnover rate compared to *A. oryzae* or *S. pombe*. *A. flavus* might recognize the splicing sites and produce AS events mainly by the intron definition (ID) mechanism owing to the high RI/CE ratio (34.75). Among the novel transcripts we were able to identify, the vast majority of transcribed ncRNAs might act as NATs to regulate gene expression at the post-transcriptional level. The transcriptional activity of *A. flavus* genes with NATs was much higher compared to genes without NATs, suggesting NATs are true transcripts rather than artifacts of leaky bidirectional transcription. Our analysis is the first global investigation of NAT function in *A. flavus*. As for DEGs, it is quite likely that the 14 up-regulated and 37 down-regulated reproduction-related genes in the sclerotia state link *A. flavus* reproduction and sclerotia development. In our ssRNA-seq data, the backbones of 38 SM pathways were transcribed in both mycelia and sclerotia states, and we identified genes linking sclerotia development and aflatoxin biosynthesis. Our data could be used to develop strategies to control aflatoxin synthesis by aflatoxigenic strains. Therefore, ssRNA-seq data provided in this study could expand our understanding of *A. flavus*.

## Supporting Information

Figure S1 **Global transcriptional profile of** ***A. flavus*** **CA43 in the mycelia state, denoted by log_2_-transformed reads count.** Coverage, the percentage of the genomic region covered by ssRNA-seq reads with a window size of 5 kb. Gene, the number of *A. flavus* genes within a window size of 5 kb. The chromosome number is shown at the left.
(TIF)

Figure S2 **GO functional enrichment analysis of** ***A. flavus*** **uORF-containing genes.** The abscissa is the percentage of genes in each GO term. The ordinate is in GO terms.
(TIF)

Figure S3 **GO functional enrichment analysis of** ***A. flavus*** **genes with NATs.** The abscissa is the percentage of genes in each GO term. The ordinate is in GO terms.
(TIF)

Table S1 Mapping summary of ssRNA-seq reads.
(XLS)

Table S2 Expression level of *A. flavus* genes and non-redundant ESTs at different development stages revealed by ssRNA-seq.
(XLS)

Table S3 The transcription status of transcriptional factors in *A. flavus* CA43 and *A. oryzae* RIB40.
(XLS)

Table S4 Novel transcripts in *A. flavus* CA43.
(XLS)

Table S5 UTRs and uORFs identified for *A. flavus* annotated genes.
(XLS)

Table S6 Alternative splicing events in *A. flavus* CA43 mycelia state.
(XLS)

Table S7 Antisense transcripts of *A. flavus* in the mycelia and sclerotia states.
(XLS)

Table S8 Genes related to asexual and sexual reproduction in *A. flavus.*
(XLS)

Table S9 Genes expressed differentially between *A. flavus* mycelia and sclerotia states.
(XLS)

Table S10 Transcriptional status of *A. flavus* secondary metabolism pathways.
(XLS)
