# Supplementary figures and images for: Characterization of Natural Antisense Transcript, Sclerotia Development and Secondary Metabolism by Strand-Specific RNA Sequencing of Aspergillus flavus
Source: PLoS One. 2014 May 21;9(5):e97814. doi: 10.1371/journal.pone.0097814 (PMC4029826; doi:10.1371/journal.pone.0097814)

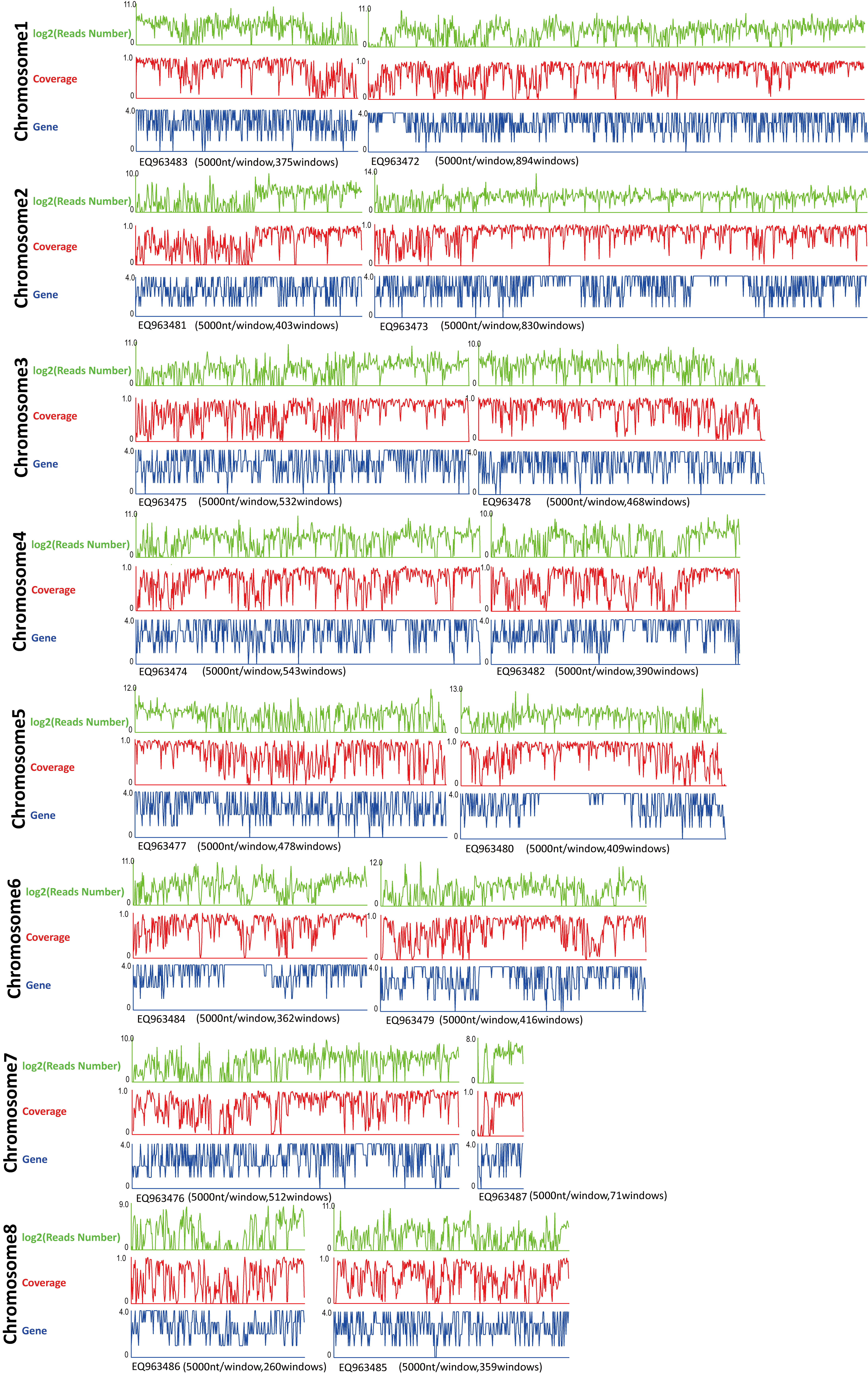

Supplement: Figure S1 — Global transcriptional profile of A. flavus CA43 in the mycelia state, denoted by log2-transformed reads count. Coverage, the percentage of the genomic region covered by ssRNA-seq reads with a window size of 5 kb. Gene, the number of A. flavus genes within a window size of 5 kb. The chromosome number is shown at the left. (TIF) [file pone.0097814.s001.tif]

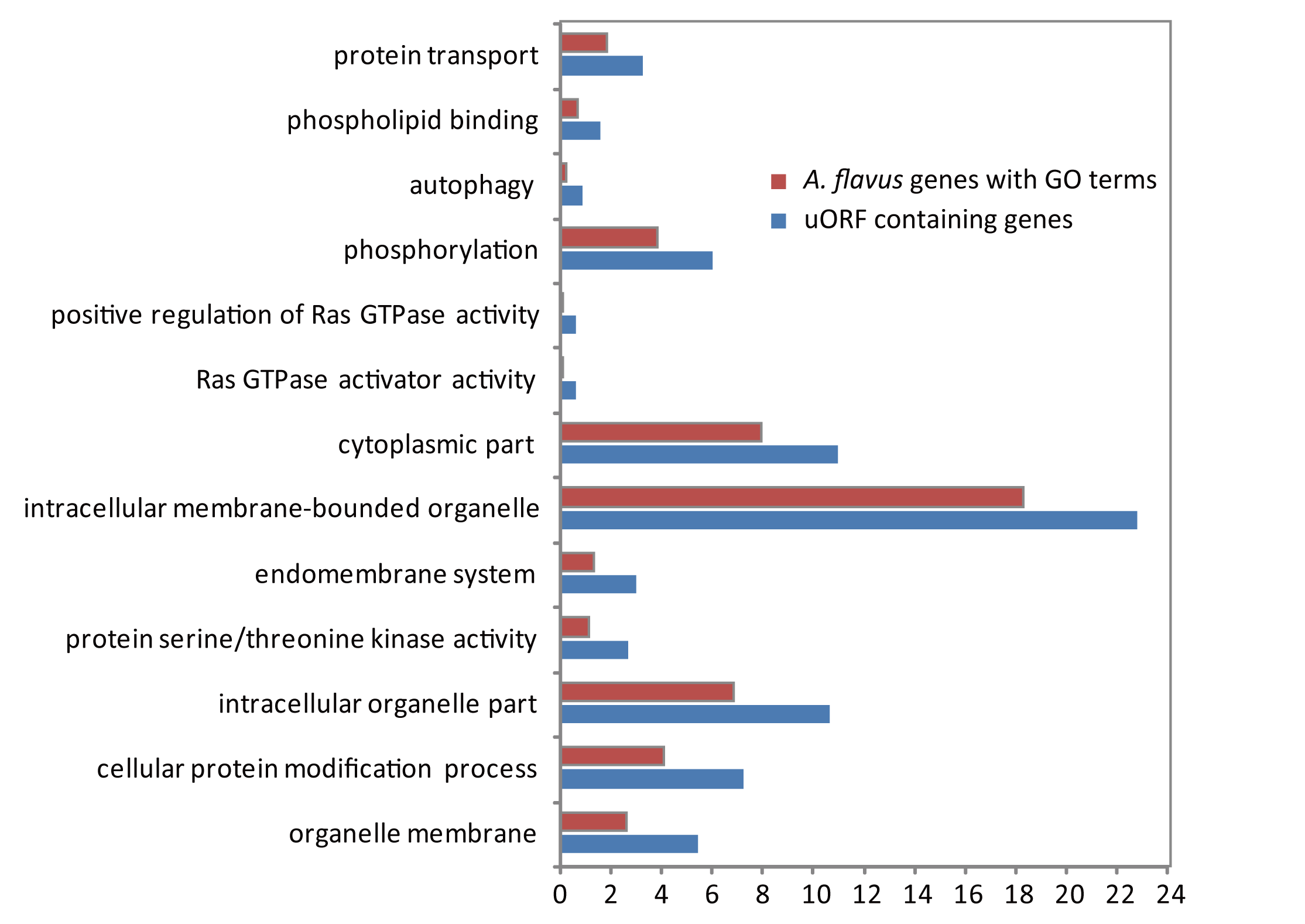

Supplement: Figure S2 — GO functional enrichment analysis of A. flavus uORF-containing genes. The abscissa is the percentage of genes in each GO term. The ordinate is in GO terms. (TIF) [file pone.0097814.s002.tif]

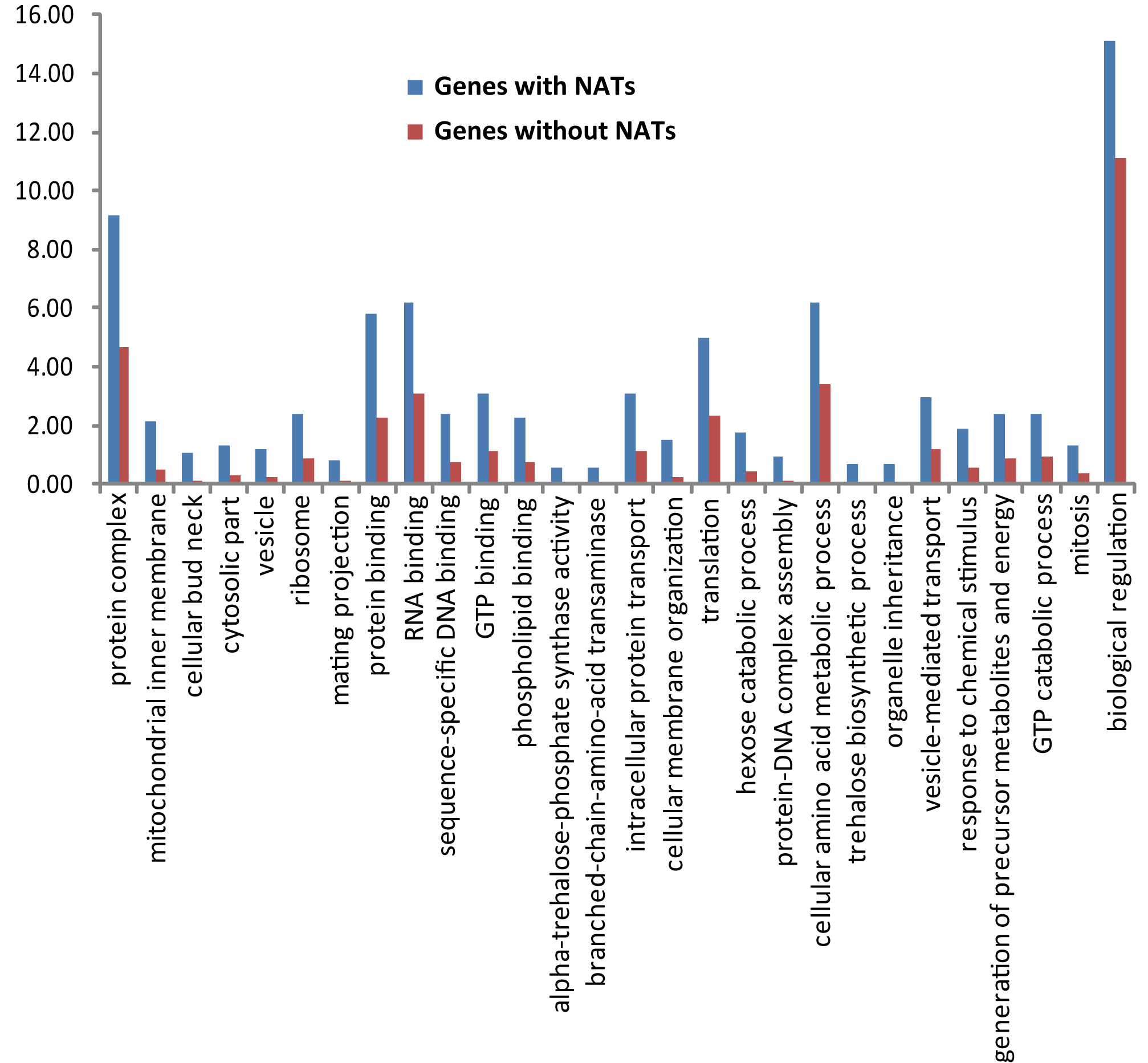

Supplement: Figure S3 — GO functional enrichment analysis of A. flavus genes with NATs. The abscissa is the percentage of genes in each GO term. The ordinate is in GO terms. (TIF) [file pone.0097814.s003.tif]
